# Effect of antioxidant therapy on the sperm DNA integrity improvement; a longitudinal cohort study

**DOI:** 10.18502/ijrm.v17i2.3987

**Published:** 2019-03-20

**Authors:** Peyman Salehi, Seyedeh Zahra Shahrokhi, Tayyebeh Kamran, Ali Ajami, Sana Taghiyar, Mohammad Reza Deemeh

**Affiliations:** ^1^Infertility center, Shahid Beheshti Hospital, Isfahan, Iran.; ^2^Department of Clinical Biochemistry, Faculty of Medical Sciences, Tarbiat Modares University, Tehran, Iran.; ^3^Department of Midwifery, Shahid Beheshti Hospital, Isfahan, Iran.; ^4^Andrology Section, Nobel Mega-lab, Isfahan, Iran.

**Keywords:** *Antioxidant*, *Reactive oxygen species*, *DNA fragmentation*, *Male infertility.*

## Abstract

**Background:**

The effect of antioxidant therapy on sperm DNA fragmentation index (DFI) and achieving natural pregnancy were under debate. Very few studies have showed the rate of pregnancy rate after the antioxidant therapy due to ethical and technical limitations.

**Objective:**

The aim of this cohort study was to determine the improvement rate of sperm DFI and natural pregnancy rate after the antioxidant therapy in infertile men.

**Materials and Methods:**

1645 infertile men were subjected for this study from May 2015 to December 2017. The Spermogram and sperm DFI were assessed using World Health Organization (WHO) 2010-based protocols and sperm chromatin structure assay (SCSA), respectively, in sperm samples before and after antioxidant therapy.

**Results:**

The total sperm DFI improvement rate was 38.9% in the total population. Sperm DFI improvement had close correlation with total motility (*r*= 0.731, p= 0.001) and progressive motility improvement (*r*= 0.885, p= 0.001); 16.8% of individuals who completed antioxidant therapy for nine months achieved natural pregnancy.

**Conclusion:**

The results of the current study suggested that SCSA along with spermogram might be a suitable option for the evaluation of fertility potential. In addition, antioxidant therapy may be useful for men with high levels of sperm DFI. However, the rate of pregnancy was still low and other treatment protocols such as assisted reproductive technology may be necessary.

## 1. Introduction

Evaluation of traditional semen parameters including concentration, motility, and morphology provided useful information about men's fertility (1). Recent assessment of human spermatozoa DNA quality may also provide additional information about fertility potential. The assessment of DNA fragmentation is one of the sperm functional tests, standardized in a clinical setting (2), for two main reasons: Firstly, there is no DNA repair system in human sperm which may increase the chance of sperm production with high levels of DNA damage for participating in fertilization process (3). The mentioned DNA damage may affect embryo development during pregnancy or it may transmit to the next generation (3, 4). Secondly, DNA fragmentation rate has a significant correlation with infertility, fertilization failure, and miscarriage rate, following assisted reproductive technology (ART) treatment as well as with natural pregnancy (4). Increasing evidence suggests that sperm chromatin structure assay (SCSA) is the most clinically available test for the assessment of chromatin integrity and DNA fragmentation (5), which shows the good scheme of chromatin status of human sperm. According to the available evidence, one of the main reasons for the increased DNA damage in human spermatozoa is Reactive Oxygen Species (ROS) (6). Several studies revealed that uncontrolled production of ROS might be an important contributor to many dysfunction and damages in DNA structure, which reduced male reproductive potential (7, 8). Considering the harmful effect of ROS on reproduction, researchers decided to treat infertile individuals with antioxidant supplements (9, 10). Several clinical studies suggested that dietary antioxidant supplements are useful to improve sperm quality and DNA integrity (11, 12). Till date, the effect of antioxidant therapy on sperm DNA Fragmentation Index (DFI) and pregnancy rate is one of the ongoing challenges toward an optimal treatment strategy. In the recent years, studies focused mainly on the effect of antioxidants on semen parameters and sperm DFI and few have evaluated pregnancy rates, because of the existence ethical and technical limitations, while evaluation of pregnancy rate should be considered as a golden purpose in clinical studies. Furthermore, the lack of precise definition of optimum dose in anti-oxidant supplements, as well as type of oral antioxidant (both alone and in combination), is a limitation of previous studies.

The present study aims to evaluate sperm DFI improvement and cumulative natural pregnancy rate after antioxidant therapy on a large population of infertile couples. Due to ethical and technical limitations, it was decided that this study be conducted without a control group in the form of a cohort study.

## 2. Materials and Methods

### Design and participants 

This longitudinal cohort study was performed at Nobel Mega-lab, Isfahan, Iran, from May 2015 to December 2017. We recruited 1645 infertile men who were referred to the andrology department for sperm analysis and SCSA program for participation in the study. Due to a large number of infertile patients referred and the difficulty and limitation in determining the control group, it was decided to conduct this study as a non-controlled cohort study. Overall, the participants were between 20 and 40 years old, and those that had a history of varicocele, surgery, and inflammation were excluded from our study. Semen samples were collected after 2–4 days of sexual abstinence. Liquefied semen were analyzed for concentration, motility, and morphology according to the 2010 World Health Organization criteria (13). Excessive semen sample prepared for SCSA test according to study design. In addition, after studies, the couples recorded data, only those couples were included in this study whose spouse had no signs of female factor infertility.

Improvement rate after antioxidant therapy was calculated using the following formula: ((subjected parameters before – subjected parameters after)/subjected parameters before) x 100.

To prevent inaccurate information, only individuals with an improvement rate higher than 15% were considered as improved person.

### Antioxidant supplement

In this study, based on the previous study, which defines cut-off value for SCSA results, 27% of sperm DFI was chosen as the cut-off score for SCSA since SCSA below 27% seems to hold chances for normal pregnancy and fertility and SCSA above 27% needs to be investigated under physicians' supervision for the assessment of fertility potential and have low chances to have a natural pregnancy (14). So, all participants who had sperm DFI greater than 27% were subjected for antioxidant therapy under the supervision of a urologist. The antioxidant supplementation contained the following active ingredients: 50 mg vitamin E (d-α-tocopherol acetate, Daro pakhsh, Iran); 500 mg vitamin C (ascorbic acid, Daro pakhsh, Iran); and 100 mg Q 10 (Daro pakhsh, Iran), which was used daily for three months. After three months, the participants repeated their spermogram and SCSA test.

### SCSA

The principles and procedure of sperm DNA fragmentation were measured by SCSA assay as previously described (15). “Briefly, Semen samples were diluted with TNE buffer and were then treated with acid detergent solution (pH= 1.2) for 30 seconds. Following this step, the samples were stained by adding 1.2 ml acridine orange as fluorescent DNA dye, which stains differently the intact and fragmented DNA. Indeed, acridine orange intercalates into double-stranded as a monomer and has green fluoresces under blue laser light (488 nm), while it acts in single-stranded DNA as an aggregate and has red fluoresces (> 630 nm). SCSA was performed on fresh ejaculated semen.”

### Flow cytometry

In total, we analyzed 10,000 sperm by FACS Calibur flow cytometer (Becton Dickinson, USA) equipped with an argon laser. Following the excitation light source, double-stranded and single-stranded DNA were detected as green and red fluorescence, respectively. An analysis of the flow cytometric data was performed using Winmdi software version 2.8.

### Ethical consideration

This study received an approval from the Institutional Review Board and ethical committee of Nobel Mega-lab, Isfahan, Iran (code: Nobel Mega-lab.Rec.1394.14), and all individuals provided a written informed consent before participation.

### Statistical analysis 

Results are reported as the means±standard deviation (SD). Statistical comparisons were performed by student's independent *t*-test. We used the Pearson's correlation and Spearman rank correlation tests to calculate the correlation coefficient and evaluate the relationship between parameters, respectively. Statistical analysis was performed using SPSS software (Statistical Package for the Social Sciences, version 21.0, SPSS Inc., Chicago, IL, USA) and values of p< 0.05 were considered as statistically significant.

## 3. Results 

**Figure 1:**
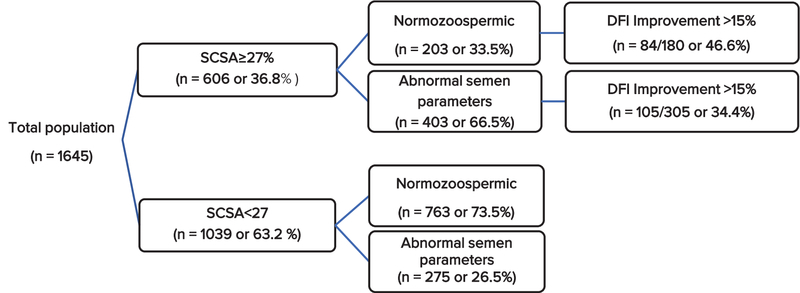
The division individual firstly based on SCSA result and then based on semen parameters and rate of SCSA improvement after antioxidant therapy; 485 individual participated in the antioxidant therapy. SCSA: Sperm Chromatin Structure Assay; DFI: DNA Fragmentation Index.

**Figure 2:**
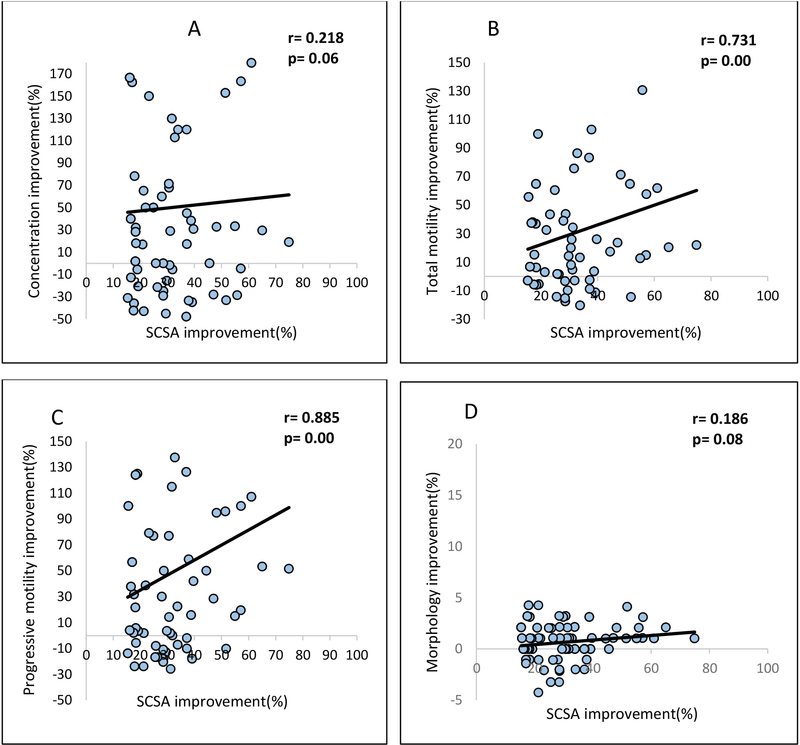
Correlation between SCSA improvement rate and A: Concentration improvement rate, B: Total motility improvement rate, C: Progressive motility improvement rate; and D: Abnormal morphology improvement rate after the antioxidant therapy in a total population.

The 1645 individuals included in the study were divided based on their SCSA-defined DNA fragmentation levels, SCSA≥ 27% (n= 606, 36.8%) and SCSA< 27 (n= 1039, 63.2%). Among individuals with SCSA≥ 27%, our result shows that 203 (33.5%) of them were Normozoospermic and 403 (66.5%) were abnormal semen parameters, whereas in SCSA< 27 group, 763 (73.5%) patients were presented as Normozoospermic and the remaining 275 (26.5%) subjects presented abnormal semen parameters as shown in Figure 1. Of 1645 individuals enrolled, 966 participants (58.7%) were labeled as Normozoospermic and the reaming 678 (41.3%) individuals as the group with abnormal semen parameters. As shown in Figure 1, the SCSA improvement rate (improvement rate higher than 15%) in Normozoospermic group and group with abnormal semen parameters were 46.6% (84/180) and 34.42% (105/305), respectively. Of course, it should be mentioned that from 606 individuals with SCSA≥ 27%, 485 patients completed the treatment period time and re-participated for spermogram and SCSA tests. The results demonstrated that 33.5% (203/606) of Normozoospermic individuals had SCSA≥ 27%, which may be corresponding to their infertility and 59.4% (403/678) of individuals with abnormal semen parameters had SCSA≥ 27%. Table I presents descriptive information for semen parameters and sperm DFI in total population, Normozoospermia group, and in groups with abnormal semen parameters before and after the treatment with antioxidant. There was a significant difference in all semen parameters in total population, Normozoospermia group, and the group with abnormal semen parameters after antioxidant therapy (p< 0.05). Figure 2 shows a correlation between SCSA improvements with other semen parameters after antioxidant therapy. We observed that SCSA result improves after antioxidant therapy has a significant correlation with total motility (r= 0.731, p= 0.001) and progressive motility (r= 0.885, p= 0.001). Table II shows the rate of cumulative natural pregnancy for nine months after beginning the study. Of course, only 321 of 530 individuals (60.56%) who were subjected to antioxidant therapy completed their information about achieving a natural pregnancy. Our result revealed that approximately 16% of individuals treated with antioxidant naturally achieved pregnancy during the treatment and follow-up.

**Table 1:** Comparison of the semen parameters and SCSA result in total population, Normozoospermic individuals and individuals with abnormal semen parameters before and after antioxidant therapy. *N* = Number of individuals; * = Considered as statically significant; SCSA: Sperm Chromatin Structure Assay.


	**Total individuals ** ***N*** **= 485**			<**Normozoospermic individuals ** ***N*** **= 180**			<**Abnormal semen parameters ** ***N*** **= 350**		
	**Before**	**After**	p**-value**	**Before**	**After**	p**-value**	**Before**	**After**	p**-value**
Total concentration(mill/ml)	41.0 ± 24.0	44.5 ± 24.1	0.003*	59.0 ± 15.0	55.0 ± 14.0	0.006*	29.0 ± 21.0	36.0 ± 25.0	0.001*
Total motility (%)	49.0 ± 17.1	56.0 ± 14.0	0.001*	64.0 ± 7.0	63.0 ± 10.0	0.002*	38.0 ± 15.0	52.0 ± 13.0	0.001*
Progressive motility (%)	37.0 ±16.0	44.0 ± 14.0	0.001*	51.0 ± 8.0	50.0 ± 10.0	0.001*	26.0 ± 13.0	42.0 ± 15.0	0.001*
Abnormal morphology (%)	95.0 ± 1.0	95.0 ± 6.0	0.027*	94.0 ± 1.0	94.0 ± 6.0	0.030*	97.0 ± 1.0	96.0 ± 5.0	0.001*
SCSA result (%)	27.3 ± 8.0	25.6 ± 7.0	0.001*	25.0 ± 7.0	23.0 ± 7.0	0.001*	29.0 ± 8.0	26.0 ± 7.0	0.001*

**Table 2:** Cumulative natural pregnancy rate nine month after the antioxidant therapy. Only 321 individuals completed their clinical cases.


	**Total population**	**Normozoospermic individuals**	**Abnormal semen parameters individuals**
Cumulative pregnancy	54/321 (16.8 %)	31/173 (17.9%)	23/148 (15.5%)

## 4. Discussion

In this study, we observed that 33.5% of Normozoospermic individuals who had the normal semen parameters showed SCSA greater than 27%, which highlights the importance of assessing DNA integrity even in Normozoospermic individuals. Given that Normozoospermic men may have an elevation of sperm DNA damage in ejaculated spermatozoa, therefore, it is necessary to caution in interpretation of the normal result of semen parameters in these patients. Indeed, a combination of spermogram test with SCSA provides useful information, which cannot be accurately obtained by spermogram alone and may help to optimize treatment strategies. There are conflicting results regarding the effect of a high sperm DFI upon semen parameters. We found that high sperm DFI has significant correlations with low sperm concentration, motility, and high abnormal morphology, as has been confirmed by previous studies (16). We observed a significant correlation between SCSA improvement rate with total and progressive motility, which provided insights that antioxidant therapy improves sperm motility and sperm DFI at the same time. This finding could perhaps be due to an existing molecular mechanism that resulted in increased sperm DNA damage and decreased sperm motility. Apoptosis as the complex molecular event may have contributed in slowing down the motility and the induction of sperm DNA damage, eventually resulting in the release of sperm with elevated levels of DNA damage and low motility (17). Several studies have reported that high levels of ROS recognized as an effective factors in male infertility are associated with sperm DNA damage, with approximately 25% of infertile men showing a high levels of semeninal ROS (7, 8). At low levels, ROS plays a significant role in many biological processes such as capacitation, hyper-activation, and acrosome reaction which is essential for successful fertilization (18, 19); however, seminal antioxidants can also protect the sperm DNA from oxidative damage (10). Excessive ROS production causes DNA damage and cell apoptosis, all of which decrease sperm numbers, motility, development of normal morphology, and consequently leads to disturbance in sperm function and fertility. In order to minimize the harmful effect of ROS, some studies have proposed that treatment with antioxidant have a positive effect on the improvement of semen quality and DNA integrity (11, 12). We evaluated semen parameters and sperm DFI before and after antioxidant therapy in men with abnormal semen parameters, Normozoospermic individuals, as well as in total population. A significant difference existed among the three groups after the antioxidant therapy in terms of semen parameters such as concentration, total motility, progressive motility, abnormal morphology and SCSA result (Table I). For a more solid and accurate conclusion, it would have been better to measure the ROS level in semen and spermatozoa directly, but due to our limitation, this was not done.

In this study, the rate of DFI improvement was more in Normozoospermia (46.6%) than the abnormal semen parameters group (34.4%), which may be unjustifiable. This data may indicate that in abnormal semen parameters individuals, the DNA damage and DFI originated from another source such as testes dysfunction or genetic disorders and cannot be treated with antioxidant therapy.

In addition to DFI and semen parameters improvement after the antioxidant therapy, many researchers proposed that improvement in pregnancy rate should be considered as the main goal of various treatment protocols. Till date, a few studies have evaluated pregnancy rates after the antioxidant therapy, and no definitive answers have yet emerged from these studies (20). On the other hand, the existence of ethical and technical issues for evaluation of pregnancy rates limits researchers to present reliable and valid data from pregnancy rates. In the present study, we followed the patients treated with the antioxidant therapy in order to evaluate cumulative pregnancy rates; however, only 321 of 530 cases completed the information about pregnancy status. Of these, 54 cases achieved natural pregnancy (16.8%) and 31 of them were Normozoospermic (17.93%) and 23 were individuals with abnormal semen parameters (15.5%). Indeed, we provided our results in form of descriptive statistic for pregnancy rate and did not have any compartment data and observed that pregnancy rate was low even after treatment with antioxidant (16.8%). For this reason, we propose other treatment protocols such as ART for overcoming infertility in these individuals. “Our study has some limitations which must be acknowledged like- (i) the lack of a control group, (ii) including only patients with sperm DFI≥ 27% for antioxidant therapy, (iii) dose used of antioxidant drug, and not evaluating the ROS levels.”

## 5. Conclusion

In conclusion, we have found that a combination of spermogram test with SCSA provides useful information for the assessment of male fertility in both Normozoospermic and abnormal semen parameters individuals. Antioxidant therapy may improve sperm DFI in both groups and the rate of improvement of sperm DFI in Normozoospermic individuals was higher than the abnormal semen parameters group (46.6% vs. 34.4%). Pregnancy rate, however, was still low (16.8%), and the use of ART treatment may be more useful for achieving pregnancy in these individuals.

##  Conflict of Interest 

All authors have seen and agreed with the contents of the manuscript and there is no conflict of interest to report.
